# Similar patterns of benzimidazole resistance alleles in ovine gastrointestinal nematodes from Western Canada and Eastern United States supports their shared origins and subsequent spread

**DOI:** 10.1016/j.ijpddr.2025.100620

**Published:** 2025-10-07

**Authors:** Camila Queiroz, Michel Levy, Russell Avramenko, Rebecca Chen, Michaela Seal, Elizabeth Redman, Anne Zajac, John Stuart Gilleard

**Affiliations:** a Faculty of Veterinary Medicine, University of Calgary, 3330 Hospital Dr, T2N4N1, Calgary, Alberta, Canada; b Department of Large Animal Clinical Sciences, Western College of Veterinary Medicine, University of Saskatchewan, 52 Campus Dr, STN5B4, Saskatoon, Saskatchewan, Canada; c Pathobiology Department, School of Veterinary Medicine, PO Box 7, Saint George's University, Grenada; d Department of Biomedical Sciences and Pathobiology, Virginia/Maryland College of Veterinary Medicine, Virginia Tech, Blacksburg, VA, 24061-0442, USA

## Abstract

Livestock movement facilitates translocation of anthelmintic resistant parasites, but the extent to which resistance emergence depends on animal movement is still poorly understood. Benzimidazole resistance is widespread in ovine trichostrongylid nematodes, and our understanding of its molecular basis now allows for molecular epidemiology investigations. This study applies deep amplicon sequencing of the isotype-1 β-tubulin locus to compare the prevalence and frequency of benzimidazole resistance Single Nucleotide Polymorphisms (SNPs), and their alleles, for trichostrongylid populations from 102 Western Canadian and 28 Eastern USA sheep flocks. For *H. contortus*, benzimidazole resistance SNPs were at fixation tin almost all flocks from both regions; that is, present at, or close to, 100 % frequency. For *T. circumcincta* and *T. colubriformis*, although at fixation in most Eastern USA flocks, resistance SNPs they were at a much lower prevalence in Western Canada, consistent with the lower anthelmintic use and selection pressure. The benzimidazole resistance SNP profiles were identical across these regions: F200Y (TTC > TAC) predominated for all three species in both regions, but there were differences between the species at codons 167 and 198. For *H. contortus*, F167Y (TTC > TAC) was at moderate prevalence but no codon 198 resistance SNPs occurred in either region. For *T. circumcincta*, E198A (GAA > GCA) was at low prevalence and for *T. colubriformis*, F200Y (TTC > TAC) was the only resistance SNP detected in both regions. Analysis of diversity and distribution of Amplicon Sequence Variants (ASVs) carrying resistance SNPs revealed that, in all three species, the same major resistance alleles were present in both regions at very similar relative frequencies. These results are consistent with a model of benzimidazole resistant ovine gastrointestinal nematodes (GIN) spreading across North America from common origins facilitated by animal movement. This model emphasizes the importance of biosecurity in limiting the emergence and spread of anthelmintic resistance in ruminant GIN. Keywords: molecular epidemiology, deep amplicon sequencing, anthelmintic resistance, nemabiome, benzimidazoles.

## Highlights

• Large-scale survey of BZ resistance SNPs in Western Canada and Eastern USA.
• Benzimidazole resistance SNPs at fixation for *Haemonchus contortus* in both regions.
• Higher USA prevalence of resistance SNPs for *T. circumcincta* and *T. colubriformis*.
• Major resistance SNPs and alleles were highly similar between the two regions.
• Data supports a shared resistance origins and emphasize animal movement relevance.

## Graphical abstract

**Figure:**
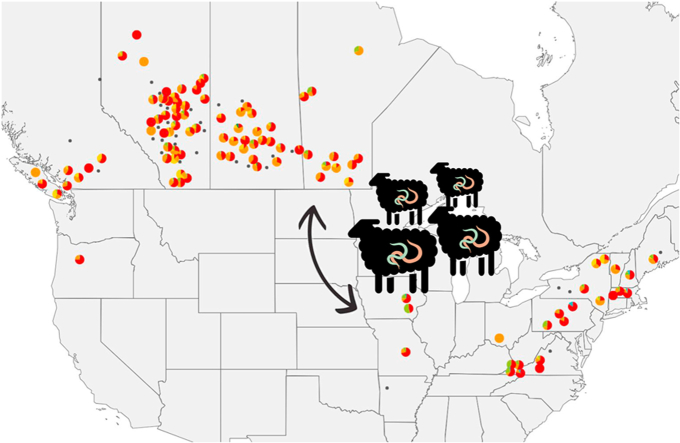

## 1 Introduction

Anthelmintic resistance has become a major limiting factor for sustainable gastrointestinal nematode (GIN) parasite control in livestock worldwide (Charlier et al., 2022). Anthelmintic resistance has been most intensively studied to date in *H. contortus,* which serves as a model for genetic studies (Gilleard, 2013). However, our understanding of the origins and spread of anthelmintic resistance is still far from complete. In the case of *H. contortus* there is increasing evidence to support the hypothesis that resistance alleles originate in a limited number of locations in a geographical region and are then spread more widely by animal movement (Chaudhry et al., 2015, 2020; Redman et al., 2015).

The benzimidazoles are a class of broad-spectrum anthelmintics with multiple members used in animal and human health including fenbendazole, albendazole, oxibendazole, oxfendazole, mebendazole and thiabendazole (Lacey, 1990; Besier et al., 2016; Gonzalez et al., 2019). This drug class was the first truly broad-spectrum group of anthelmintics for livestock GIN parasite control and has now been used in small ruminants for around 60 years (Brown et al., 1961; Gordon, 1961). It was also the first drug class for which resistance was reported in *H. contortus*, which has now become globally widespread in this parasite species (Drudge et al., 1964). The benzimidazoles are also the anthelmintic drug class for which the genetic basis of resistance is best understood (Gilleard, 2006; Gilleard and Redman, 2016). They are believed to act by binding to β-tubulin monomers, and so inhibiting microtubule polymerisation (Lacey, 1988, 1990; Chai et al., 2021). The importance of the β-tubulin drug target in benzimidazole resistance was first demonstrated functionally in nematodes by mutagenesis experiments in *Caenorhabditis elegans* in which all resistance loci mapped to the *ben*-1 β-tubulin locus (Driscoll et al., 1989). The relevance of this to parasitic nematodes was then confirmed by demonstrating that different alleles of the *H. contortus* isotype-1 β-tubulin gene could modulate the benzimidazole sensitivity of *C. elegans* by heterologous expression (Kwa et al., 1995). Subsequent to this work, a number of SNPs in three different codons of the isotype-1 β-tubulin gene of *H. contortus,* or related ruminant GIN parasite species of the superfamily trichostrongyloidea, have been associated with benzimidazole resistance: F200Y (TTC > TAC) (Kwa et al., 1994; Elard and Humbert, 1999), F167Y (TTC > TAC) (Silvestre and Cabaret, 2002), E198A (GAA > GCA) (Ghisi et al., 2007), E198L (GAA > TTA) and E198V (GAA > GTA) (Redman et al., 2015; Avramenko et al., 2019; Martínez-Valladares et al., 2020; Mohammedsalih et al., 2020). CRISPR cas-9 genome editing has confirmed that all these SNPs confer similar levels of phenotypic benzimidazole resistance in *C. elegans* (Dilks et al., 2020).

Various studies have reported the occurrence of these benzimidazole resistance-associated SNPs at codons 167, 198 and 200 in a variety of trichostrongylid species of cattle and sheep from different geographical locations. The F200Y (TTC > TAC) SNP is the most common SNP in *H. contortus* and other trichostrongylid nematodes that have been studied. For example, it has been reported as the most predominant benzimidazole resistance SNP in Brazil (Nunes et al., 2013; Santos et al., 2014), India (Chandra et al., 2014; Chaudhry et al., 2015), Pakistan (Chaudhry et al., 2016), China (Zhang et al., 2016), Ireland, Italy, Switzerland (Ramunke et al., 2016), Mexico (Encalada-Mena et al., 2014), United States (Chaudhry et al., 2014, 2020) and the UK (Redman et al., 2015). However, the other resistance SNPs occur to varying extents in different regions. For example, the next most common SNP, F167Y (TTC > TAC), has been reported in the UK (Redman et al., 2015; Avramenko et al., 2019), Ireland, Italy, Switzerland (Ramunke et al., 2016), France (Silvestre and Cabaret, 2002), Germany (Demeler et al., 2013), the United States (Chaudhry et al., 2020) and Canada (Barrère et al., 2013b). In fact, F167Y (TTC > TAC) was reported at a higher overall frequency than F200Y (TTC > TAC) in *H. contortus* from sheep in the UK (Avramenko et al., 2019) and in Northeast Brazil (Lamberta et al., 2017; Santos et al., 2017). The E198A (GAA > GCA) SNP has been found in several countries, including India (Chaudhry et al., 2015), China (Zhang et al., 2016), Germany (Demeler et al., 2013), Ireland, Italy, and Switzerland (Ramunke et al., 2016). Most recently, the E198L (GAA > TTA) multiple nucleotide polymorphism (MNP) has been described in *T. circumcincta* in the UK (Redman et al., 2015; Avramenko et al., 2019) and Spain (Martínez-Valladares et al., 2020) and in *H. contortus* in Sudan (Mohammedsalih et al., 2020). Interestingly, although the E198L (GAA > CTA) SNP has been reported more rarely than for the other two codons to date, in one Spanish study it was present at high frequency in flocks (10.7–80.7 % before and 82.3–92.8 % after benzimidazole treatment) and was the only isotype-1 β-tubulin resistance SNP resistance detected in benzimidazole resistant *T. circumcincta* populations. An E198V (GAA > GTA) SNP was also described, albeit at very low frequencies, in the same populations in which the E198L (GAA > TTA) SNP was present in the previously cited studies (Martínez-Valladares et al., 2020). Finally, E198K (GAA > AAA), E198I (GAA > ATA) and E198∗ (stop codon) (GAA > TAA) were recently described in *H. contortus* populations from Sudan (Mohammedsalih et al., 2020), in addition to E198G (GAA > GGA) in Australia (Francis and Šlapeta, 2023; Francis et al., 2024) and E198K (GAA > AAA) in Uganda (Kalule et al., 2023). In summary, the prevalence and distribution of the different isotype-1 β-tubulin benzimidazole resistance SNPs s varies between sheep GIN species and geographical regions. As such data accumulates, it will be increasingly possible to undertake molecular epidemiological studies to help us better understand the patterns of resistance origins and spread.

Deep amplicon sequencing is now well established to screen for benzimidazole resistance SNPs in the isotype-1 β-tubulin gene, and examine their allelic diversity, for the major trichostrongylid gastrointestinal nematode species of livestock making larger-scale molecular epidemiology studies feasible (Avramenko et al., 2019). In this study, we have undertaken a large-scale study of benzimidazole resistance SNPs and alleles of ovine GIN in two geographically separate regions of North America: Western Canada and Eastern USA. We surveyed the three most common ovine GIN species present - *H. contortus, T. circumcincta* and *T. colubriformis* – in 102 Western Canadian and 28 Eastern USA sheep flocks. We report a high degree of similarity in the benzimidazole resistance SNPs and ASVs (resistance alleles) present for all three parasite species across the two regions, suggesting the origins of benzimidazole resistance are shared between these two distinct regions of North America.

## 2 Materials and methods

### 2.1 Sample collection, fecal egg counting, harvesting and hatching

**Fig. 1:**
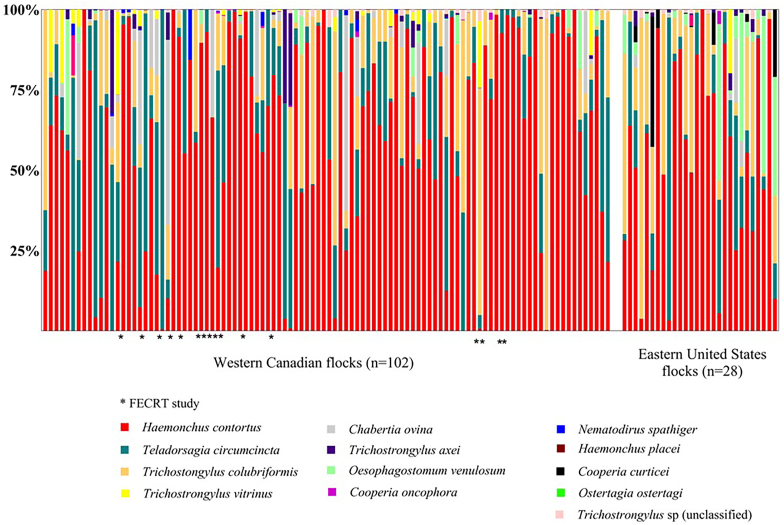
**Species composition of gastrointestinal nematode communities in sheep flocks in Western Canada and Eastern USA determined by ITS2 nemabiome metabarcoding**. Canadian samples were collected as part of another study conducted from 2014 to 2019. From 102 sheep flocks, 86 were sampled by producers and 16 samples were collected as part of a Fecal Egg Count Reduction Test study, and are identified with ∗ (Queiroz et al., 2020). United States samples were pooled from 4 to 7 animals from 28 flocks. Relative abundance of gastrointestinal nematode species in ewes from 102 Western Canadian and 28 Eastern United States sheep flocks was determined by ITS2 rDNA nemabiome metabarcoding of first stage larvae in pre-treatment samples.

**Supplementary Fig. 1:**
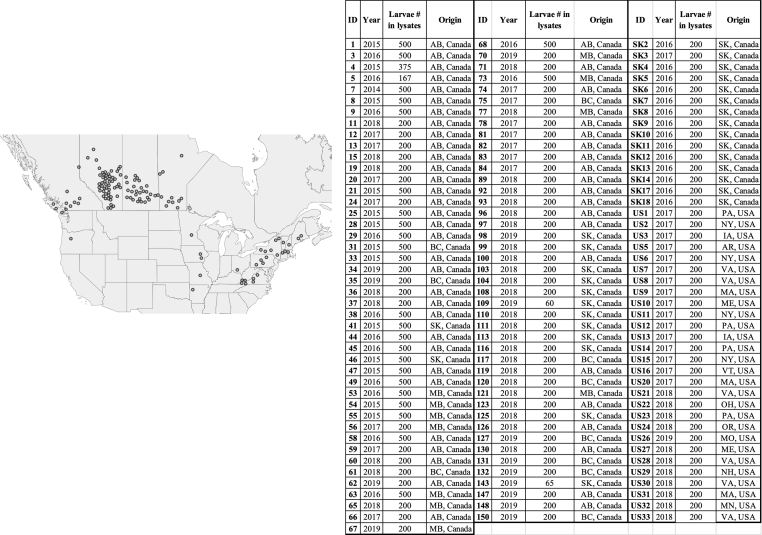
**Regional distribution and information of samples obtained from Western Canada (collected as part of**Queiroz et al., 2020**) and Eastern United States.** Map on the left shows approximate geographical locations of farms sampled from Western Canada (n = 102) and Eastern United States (n = 28). Table on the right shows information on samples ID, year of collection, number of larvae included in lysate preparation, and origin of the sample (State and Country).

The Canadian samples used in this work were collected and archived as part of a project undertaken in Western Canada from 2014 to 2019 (Queiroz et al., 2020). A total of 102 sheep farms; 10 from British Columbia (BC), 52 from Alberta (AB), 29 from Saskatchewan (SK) and 11 from Manitoba (MB) were included in the work described here (Supplementary Fig. 1). For 86/102 Canadian farms, samples were collected by producers by gathering 20 ewes that had not been dewormed for at least 8 weeks. Fecal samples were collected from ewes either *per rectum* or from the ground whilst still fresh and sent to the laboratory by courier in sealed plastic bags. For the remaining 16/102 Canadian farms, fecal samples were collected by the research team during farm visits as part of a previous Fecal Egg Count Reduction Test (FECRT) study (marked with ∗ on Fig. 1) (Queiroz et al., 2020). For this study, we only included samples collected before anthelmintic treatment. Twenty-eight GIN populations from commercial sheep flocks in the United States were collected and processed at Virginia Tech College of Veterinary Medicine. These samples consisted of pools from 4 to 7 animals, collected from 2017 to 2019, from multiple locations of United States (Supplementary Fig. 1).

Each year, as fecal samples were collected over the summer, fecal egg counts were completed within 24h of sampling/receiving in the laboratory, followed by immediate harvest of eggs, which were then incubated for 24–48h for hatching. Larvae were then counted, fixed in 70 % Ethanol and stored at 4 °C until used. When farm visits and sample receiving was completed by the end of the summer, the research team then started DNA extractions. All DNA lysates were prepared, together with 1:10 dilutions, and all neat and diluted lysates were stored at −80 °C. After all samples were processed, usually by the end of Fall, we would start preparing PCR and amplicon sequencing. Details on these steps are provided below.

A modified McMaster method (Paracount-EPG™, Chalex, LLC) with sensitivity of 16.66 epg was used to perform egg counts on all fecal samples. For the producer-applied treatment samples, 6 g of all 20 individual samples were pooled from each farm, and the overall FEC determined as the mean of four independent aliquots taken and counted from each pool (Queiroz et al., 2020). For the FECRT study samples, FEC had been performed on 20 individual ewes in the group, before any anthelmintic treatment. For subsequent harvesting of eggs for deep amplicon sequencing, 2 g of each individual fecal sample were pooled and thoroughly mixed prior to processing for larval recovery.

Strongyle eggs were recovered following the protocol described previously (Queiroz et al., 2020). Briefly, a 24 g aliquot of the pooled samples was thoroughly mixed with 13 % sodium chloride solution (1.06 gravity) and washed through a coarse kitchen sieve. The egg-containing filtrate was submitted to four serial centrifugations at 3600 G, alternating additions of distilled water and 13 % sodium chloride solution, to sediment and float the eggs, respectively, while cleaning debris. The final pellet containing the nematode eggs was resuspended in distilled water and washed through a 20 μm sieve to clean the remaining debris and retain the eggs. The eggs were then rinsed from the sieve and incubated in tap water at 22 °C for 24–48 h to hatch the eggs. First stage larvae (L1) were then harvested and fixed in 70 % ethanol and stored at 4 °C until use.

### 2.2 DNA lysate preparation from first stage larvae (L1)

For the Canadian samples, we initially used approximately 500 first stage larvae (L1) from samples from the earlier years of the project (2014–2016). For samples collected in 2017–2019, lysates were prepared from approximately 200 larvae from each sample to maximize the number of samples that could be included in the study. Approximately 200 larvae were aliquoted from each of the United States samples. On three samples, the total number of larvae was less than 200 (samples 5, 109 and 143, with 167, 60 and 65 larvae respectively), therefore the lysates were prepared from all the larvae harvested from the sample.

Ethanol-fixed larvae from each sample were washed three times by centrifugation with lysis buffer made in-house (50 mM KCl, 10 mM Tris, pH 8.3, 2.5 mM MgCl_2_, 0.45 % Nonidet p-40, 0.45 % Tween 20, 0.01 % gelatin) to remove all ethanol. The washed pellet was resuspended in 50 μL lysis buffer and placed at −80 °C. Lysis was achieved by adding 6 μL 20 mg/mL proteinase K (Thermo Scientific) and heating at 55 °C for 120 min, followed by 95 °C for 20 min to inactivate proteinase K. Once lysed, 1:10 dilutions were made with molecular grade water to use as PCR template and the remaining original neat lysate (undiluted) was archived at −80 °C.

### 2.3 ITS2 nemabiome metabarcoding and deep amplicon sequencing of the isotype-1 β-tubulin gene

ITS2 nemabiome metabarcoding refers to the amplicon deep sequencing of the ITS2 rDNA marker to identify, and determine the relative proportions of, the trichostrongylid species present in each sample. It was performed using previously described methods and further information is available at www.nemabiome.ca (Avramenko et al., 2015; Redman et al., 2019). For deep amplicon sequencing of the isotype-1 β-tubulin gene four previously described primer sets were used to PCR amplify a region of the gene encompassing codons 167, 198 and 200 from multiple ovine and bovine trichostrongylid nematode species (Avramenko et al., 2019). PCR conditions were 5 μL 5x NEB Q5 Reaction Buffer (New England Biolabs Ltd), 0.5 μL 10 mM dNTPs, 1.25 μL 10 μM Forward primer mixture, 1.25 μL 10 μM Reverse primer mixture, 0.25 μL NEB Q5 polymerase, 10.75 μL molecular grade water and 6 μL DNA 1:10 lysate dilution. The thermocycling parameters were 98^o^ C for 30 s, followed by 38 cycles of 98^o^ C for 10 s, 65^o^ C for 15 s, and 72^o^ C for 25 s, followed by 72^o^ C for 2 min. PCR products were purified with AMPure XP Magnetic Beads (1×) (Beckman Coulter, Inc), following manufacturer's protocol. All samples were eluted in 32 μL of molecular grade water (Avramenko et al., 2015, 2019).

Following purification of the first round PCR amplicons, Illumina barcode indices and P5/P7 sequencing regions were added to amplicons using limited-cycle PCR amplification, using indices contained in the Nextera XT Index Kit v2 set (Oligonucleotide sequences © 2016 Illumina, Inc.). Indices were combined to make 384 unique forward and reverse barcode combinations per run (total of four 96-well plates per run). PCR conditions were 5 μL 5X KAPA HiFi HotStart Fidelity Buffer (KAPA Biosystems, USA), 1.25 μL Forward Primer (S502-S522) (10 μM), 1.25 μL Reverse Primer (N701-N729) (10 μM), 0.75 μL dNTPs (10 mM), 0.5 μL KAPA HiFi HotStart Polymerase (0.5 U), 13.25 μL molecular grade dH_2_O, and 3 μL of first round purified PCR product as template. Thermocycling parameters were 98^o^ C for 45 s, followed by nine cycles of 98^o^ C for 20 s, 63^o^ C for 20 s, 72^o^ C for 2 min. Amplicons were purified with AMPure XP magnetic beads (1×) following the steps described previously (Avramenko et al., 2019).

The DNA concentration of each individual library was determined using the NanoVue plus Spectrophotometer (GE). A 50-ng aliquot of each purified library was used to create a pooled library, the final concentration of which was determined using the KAPA qPCR Library Quantification Kit (KAPA Biosystems, USA). The library was then diluted to 4 nM and run on an Illumina Desktop Sequencer using a 500-cycle paired-end reagent kit (MiSeq Reagent Kit, v2, MS-103-2003) at a final concentration of 15 pM, with the addition of 25 % 15 pM PhiX control v3 (Illumina, FC-110-3001). Samples were de-multiplexed using the MiSeq onboard software based on the index combinations. All generated sequences are publicly available on the Sequence Read Archive (SRA - PRJNA1178696).

### 2.4 Isotype-1 β-tubulin Amplicon Sequence Variant (ASV) generation and filtering

Forward and reverse sequences obtained from each sample were analyzed using a bespoke bioinformatics pipeline. Sequences were first merged in mothur software v.1.41.0 (Schloss et al., 2009) to make single contigs and filtered to remove reads shorter than 200 bp and longer than 450 bp. Sequences were aligned to a trichostrongylid nematode isotype-1 β-tubulin database described in (Avramenko et al., 2019) using default Needleman-Wunsch algorithm and filtered to a minimum similarity of 60 % of at least 10 % of any reference sequence. Sequences were then taxonomically classified using the k-nearest neighbor method with k = 3. A list with sequences assigned to each species from all samples was created and used to divide the original raw forward and reverse FASTQ sequences into individual FASTQ files for each species from each sample using the software Seqtk v.1.3 (https://github.com/lh3/seqtk).

Primers were detected and removed from classified sequences using default parameters of Cutadapt version 2.10 (Martínez-Valladares et al., 2020) with a 20 % error rate, i.e. a primer with sequence length 20bp was detected and removed if the number of bp mismatch is below 4. Reads were removed if both the forward and reverse primers were not detected. Sequences were then analyzed using dada2 version 1.14.0 (Callahan et al., 2016) on R version 3.6.3, to count the number of times each unique Amplicon Sequence Variant (ASVs) is observed in each sample. Initially, quality filtering was performed using default parameters in filterAndTrim() function, but with a maximum expected error threshold maxEE = 1 for both forward and reverse reads, in which reads with an expected error rate greater than 1 were discarded. The algorithm then samples sequences up to 100 million bases to learn the error rate in the data, using default parameters in learnErrors() function, and finally forward and reverse reads were merged and chimeric sequences removed after error-correction using dada(), mergePairs(), and removeBimeraDenovo() functions. Sequences obtained in different runs were processed separately to account for errors originated from the sequencing run. Finally, the algorithm identified unique ASVs, creating an ASV table with the number of times each ASV was observed in each sample. ASV tables from different sequencing runs were merged.

The aim of the work was to focus on the major resistance alleles identified in the different populations and to avoid potential artifactual ASVs. Consequently, ASVs with less than 30 reads in at least 4 individual samples and were filtered out to exclude potential artifacts for the final dataset analyzed (see Supplementary Tables 1, 2, and 3).

### 2.5 Statistical analysis, haplotype network analysis and geospatial mapping

Results were sorted by year, drug treatment, and farm ID and imported to GraphPad Prism 8 for macOS version 8.1.0, April 3, 2019 (GraphPad Software, San Diego, California USA, www.graphpad.com), where Kruskal-Wallis, Mann-Whitney, Spearman correlation statistical tests were performed as appropriate and as described in the results section and on legends of each figure and table.

For the haplotype network analysis, multiple sequence alignment was applied to the filtered ASV sequences using G-INS-i algorithm (--globalpair --maxiterate 1000) in MAFFT version 7.526 (Katoh and Standley, 2013). Finally, the R package pegas (Paradis, 2010) version 1.3 was used to infer evolutionary relationship between ASVs, building a statistical parsimony haplotype network (Templeton et al., 1992) from a pairwise character difference distance matrix calculated with R package haplotypes (https://cran.r-project.org/web/packages/haplotypes/index.html) version 1.1.3.1 distance() function, using “sic” (simple indel coding) method where continuous gap characters “-” were treated as one state change.

To generate geospatial maps with the SNP frequency distributions, latitude and longitude information were obtained from postal codes of the farms sampled. To ensure confidentiality of the farm locations, we applied spatial jittering using jittter() function from base R (version 4.4.2), with parameters factor = 1, amount = 1. This function adds a small random noise to each geographical coordinate, preventing exact identification of true farm locations. The approach is widely used to balance data confidentiality with spatial accuracy. The jittered coordinates were then visualized with R package leaflet (version 2.2.2). When necessary, some points were manually and minimally moved to prevent overlap between farms using R package leaflet.extras (version 2.0.1) (https://cran.r-project.org/web/packages/leaflet.extras/index.html). Data visualization was developed in R software (R version 4.4.2) to analyze geospatial distribution of anthelmintic resistance of the three most prominent GIN species of the dataset.

Interactive maps built from R package leaflet version 2.2.2 (Cheng et al., 2025) were used to create geospatial visualization of anthelmintic resistance frequency patterns. Each SNP (F200Y, E198L, F167Y) present per species was visualized by mapping the frequency gradient for that SNP. The proportion of resistant and susceptible haplotypes per sample were visualized as pie charts across regions sampled using R package leaflet.minicharts version 0.6.2 (https://cran.r-project.org/web/packages/leaflet.minicharts/index.html). Canada and US administrative boundaries used in visualizations were fetched from geoBoundaries (Runfola et al., 2020) using rgeoboundaries R package version 1.2.9 (https://github.com/wmgeolab/rgeoboundaries) and simplified using R package sf version 1.0–16 (Pebesma, 2018) st_simplify() function and dTolerance = 3000 and preserveTopology = FALSE settings.

## 3 Results

### 3.1 Species composition of gastrointestinal nematode communities in sheep flocks from Western Canada and Eastern USA

ITS2 nemabiome metabarcoding was performed on L1 populations pooled from 102 Western Canadian (20 individual ewe fecal samples per flock) and 28 Eastern USA sheep flocks (4–7 individual ewes per flock) (Fig. 1). Overall, the parasite species present were similar, with *H. contortus* being most abundant overall followed by *T. circumcincta* and *T. colubriformis* in both regions. *C. curticei* was detected in several US but not Canadian flocks. There was no significant difference in alpha diversity (Shannon Index) between Western Canada and United States (Wilcoxon rank-sum test, W = 540, p = 0.4826). The average Bray-Curtis dissimilarity (beta-diversity) between USA and Canada samples was 0.483. PERMANOVA analysis indicated no statistically significant difference in species composition between the two populations (F = 2.304, R^2^ = 0.017, df = 1,130, p = 0.0780). The observed moderate differences in species composition between the two regions are likely influenced by the discrepancy in sample numbers or other geographic factors.

### 3.2 Comparison of the isotype-1 β-tubulin benzimidazole resistance SNPs present in *H. contortus, T. circumcincta* and *T. colubriformis* populations between Western Canadian and Eastern USA sheep flocks

**Fig. 2:**
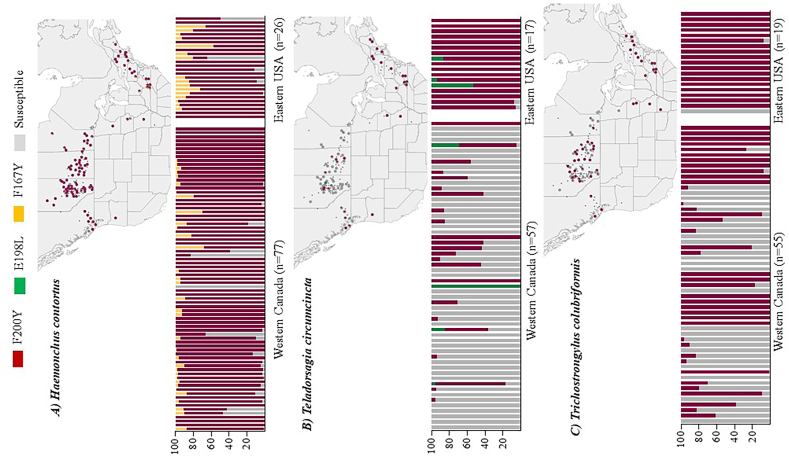
**Geographical distribution of canonical codon 167, 198 and 200 isotype-1 β-tubulin benzimidazole resistance SNPs in *Haemonchus contortus, Teladorsagia circumcincta* and *Trichostrongylus colubriformis* from Western Canadian and Eastern United States sheep flocks.** Deep amplicon sequencing was performed on genomic DNA isolated from pooled gastrointestinal nematode L1 populations of 102 Western Canadian and 28 Eastern USA sheep flocks to determine isotype-1 β-tubulin benzimidazole resistance SNP frequencies for *Haemonchus contortus* (panel A), *Teladorsagia circumcincta* (panel B) and *Trichostrongylus colubriformis* (panel C). The isotype-1 β-tubulin mapped read depth for individual species had a range of 200-37,000 reads per sample (mean 3800) and the ASVs were filtered to only include those represented by least 30 reads in at least 4 separate samples in the final dataset. For each of the three major trichostrongylid nematode species the upper panel shows the geospatial distribution of the detected canonical isotype-1 β-tubulin BZ resistance SNPs as pie charts displaying the relative SNP frequencies in each population and the lower panel shows the same data as bar plots.

Targeted deep amplicon sequencing was performed on pooled L1 populations from ewes for Western Canadian and Eastern USA sheep flocks to determine isotype-1 β-tubulin benzimidazole resistance SNP frequencies for the three most common gastrointestinal nematode species (Fig. 2). F200Y (TTC > TAC) was the resistance SNP with the highest prevalence and overall frequency for all three GIN species in both regions (Fig. 2).

**Supplementary Fig. 2:**
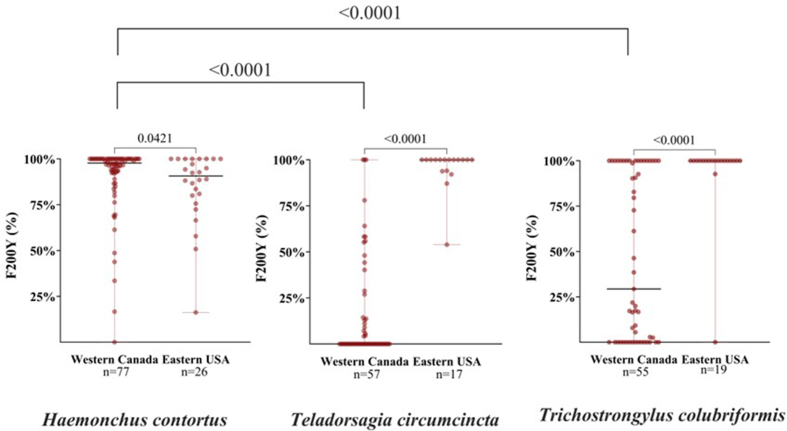
**Comparison of isotype-1 β-tubulin F200Y (TTC > TAC) SNP frequencies in *Haemonchus contortus, Teladorsagia circumcincta* and *Trichostrongylus colubriformis* between Western Canadian and Eastern United States sheep flocks.** The frequencies of the isotype-1 β-tubulin F200Y (TTC > TAC) SNP in *Haemonchus contortus*, *Teladorsagia circumcincta* and *Trichostrongylus colubriformis* were plotted for Western Canada and Eastern USA and the statistical significance of the differences between the two regions was determined by the Mann-Whitney test using GraphPad Prism version 8.1.0 for macOS, GraphPad Software, San Diego, California USA, www.graphpad.com. Solid lines represent the median and range of F200Y (TTC > TAC) frequencies in *H. contortus, T. circumcincta* and *T. colubriformi*s.

In the case of *H. contortus,* the F200Y (TTC > TAC) SNP was present at very high frequencies, indeed at fixation, on almost all farms in both Western Canada and Eastern USA. It was present at a prevalence of 76/77 flocks and mean overall frequency of 91 % in Western Canada and a prevalence of 26/26 flocks and mean overall frequency of 85 % in Eastern USA (Fig. 2A and Supplementary Fig. 2). The F167Y (TTC > TAC) SNP was also present on many farms in both regions, but at a lower prevalence and much lower frequencies; a prevalence of 36/77 flocks and mean overall frequency of 3.5 % in Western Canada and a prevalence of 16/26 flocks and mean overall frequency of 9.8 % in Eastern USA). No resistance SNPs at codon 198 were detected in *H. contortus* in either region.

In the case of *T. circumcincta*, the F200Y (TTC > TAC) SNP was also the most prevalent resistance SNP in both Western Canada and Eastern USA (Fig. 2B). However, unlike for *H. contortus,* it was present at a much higher prevalence and frequency in the Eastern USA (17/17 farms and 95 % mean frequency) than in Western Canada (23/57 farms and 17 % mean frequency) in this nematode species (Fig. 2B and Supplementary Fig. 2). A E198L (GAA > TTA) SNP was present in 4/57 farms in Western Canada (3 % mean frequency) and in 3/17 farms in Eastern United States (4 % mean frequency). The F167Y (TTC > TAC) SNP was not detected in *T. circumcincta* in either region.

**Supplementary Fig. 3:**
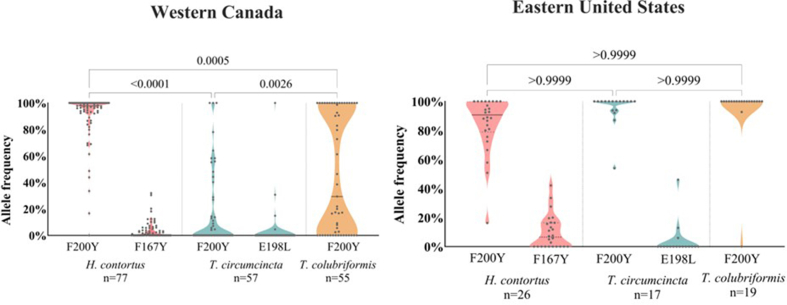
**Comparison of the isotype-1 β-tubulin codon 167, 198 and 200 BZ resistance SNP frequencies in *H. contortus*, *T. circumcincta* and *T. colubriformis* in Western Canada and Eastern USA.** Violin plots show the frequency of F200Y (TTC > TAC), E198L (GAA > TTA), and F167Y (TTC > TAC) on isotype-1 β-tubulin SNP frequencies for *Haemonchus contortus*, *Teladorsagia circumcincta* and *Trichostrongylus colubriformis* from 102 Western Canadian and 28 Eastern USA sheep flocks. Different species are represented by different colors; *H. contortus* (pink)*, T. circumcincta* (teal) and *T. colubriformis* (orange) and gray dots represent SNP frequencies in each flock. Dotted lines represent quartiles, and solid lines represent median of each violin plot. The total frequency of SNPs is shown after filtering out ASVs that did not fit the criteria of at least 30 reads in at least four samples. Statistical significance of the differences between the groups was determined by Kruskal-Wallis test followed by Dunn's multiple comparisons test using GraphPad Prism version 8.1.0 for macOS, GraphPad Software, San Diego, California USA, www.graphpad.com.

In the case of *T. colubriformis,* F200Y (TTC > TAC) was again the most common resistance SNP in both Western Canada and Eastern USA (Fig. 2C) and like *T. circumcincta*, it was present much at a higher prevalence and overall frequency in the USA (18/19 farms and 94 % mean frequency) than in Canada (39/55 farms and 48 % mean frequency) (Fig. 2C and Supplementary Fig. 2). This was the only benzimidazole resistance SNP detected in either region for this species. The frequencies of the different resistance SNPs in the different species for each flock in the two regions is summarized in Supplementary Fig. 3.

### 3.3 Comparison of isotype-1 β-tubulin Amplicon Sequence Variants (ASVs) from *H. contortus*, *T. circumcincta* and *T. colubriformis* between Western Canada and Eastern USA sheep flocks

**Fig. 3:**
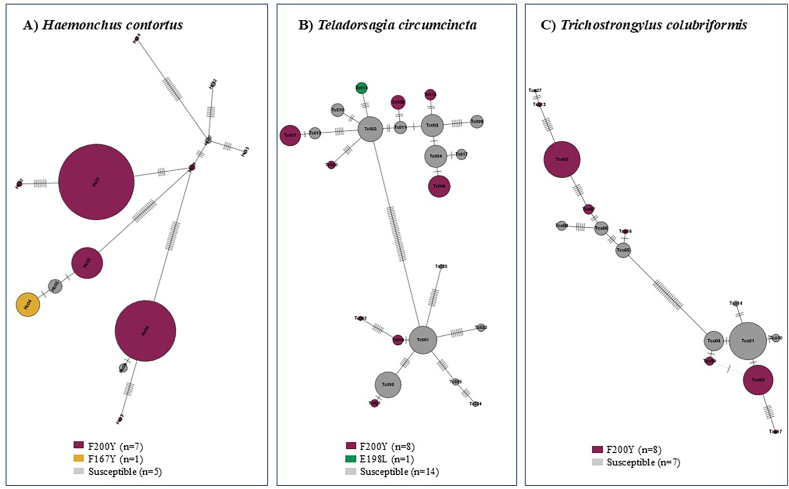
**Statistical parsimony haplotype networks of isotype-1 β-tubulin ASVs from Western Canadian and Eastern United States sheep flocks indicating the presence of codon 200, 198 and 167 benzimidazole resistance SNPs in A) *Haemonchus contortus,* B) *Teladorsagia circumcincta* and C) *Trichostrongylus colubriformis*.** Isotype-1 β-tubulin gene Amplicon Sequence Variants (ASVs) were analyzed using a statistical parsimony haplotype network, which minimizes the total number of evolutionary steps required to explain the dataset (Templeton et al., 1992). Each circle represents a unique haplotype. The color of each circle represents the absence (gray = susceptible) or presence of a benzimidazole resistance-associated SNPs, being dark red (F200Y (TTC > TAC)), green (E198L (GAA > TTA)), and yellow (F167Y (TTC > TAC)). The size of each circle represents the relative frequency of the haplotype in the dataset. Short crossing lines represent the number of SNPs between adjacent haplotypes. Panel A; *Haemonchus contortus,* Panel B: *Teladorsagia circumcincta* and Panel C: *Trichostrongylus colubriformis*. The network uses linear scale for the circle size representing haplotype frequencies top show the main ASVs. ASVs represented have been filtered for the presence of at least 30 reads in at least 4 samples.

**Fig. 4:**
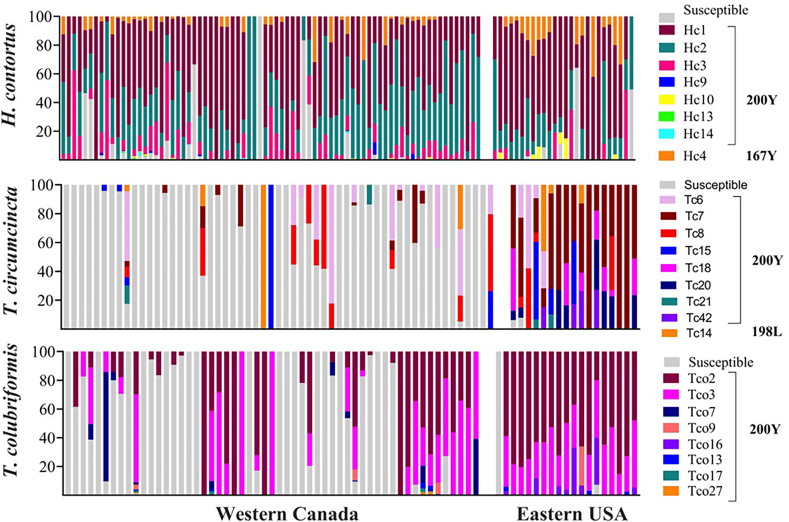
**Comparison of the frequencies of *Haemonchus contortus, T. circumcincta* and *T. colubriformis* isotype-1 β-tubulin ASVs which carry canonical codon 200, 198 and 167 benzimidazole resistance SNPs in Western Canadian and Eastern United States sheep flocks.** Deep amplicon sequencing was performed on genomic DNA isolated from pooled gastrointestinal nematode L1 populations of 102 Western Canadian and 28 Eastern USA sheep flocks to determine isotype-1 β-tubulin benzimidazole resistance mutation frequencies for *Haemonchus contortus* (panel A), *Teladorsagia circumcincta* (panel B) and *Trichostrongylus colubriformis* (panel C). 200–500 L1 larvae were isolated from a pool of 20 individual ewe fecal samples per flock for each genomic DNA preparation. The ASVs used to generate the resistance SNP frequencies were filtered to only include those represented by least 30 reads in at least 4 separate samples in the final dataset.

**Fig. 5:**
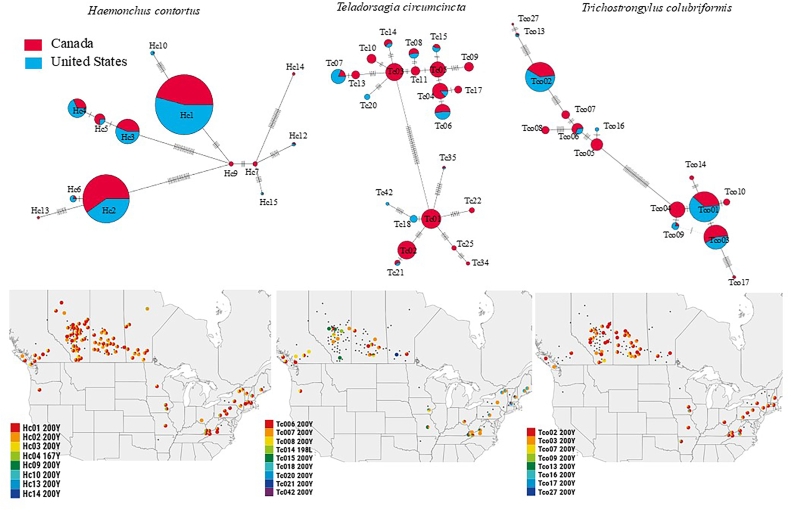
**Geographical distribution of the F200Y (TTC > TAC) ASVs in *Haemonchus contortus*, *Teladorsagia circumcincta* and *Trichostrongylus colubriformis.*** Isotype-1 β-tubulin gene Amplicon Sequence Variants (ASVs) were analyzed using a statistical parsimony haplotype network, which minimizes the total number of evolutionary steps required to explain the dataset (Templeton et al., 1992). Each circle represents a unique haplotype. Colors inside each pie chart represent each region in which the haplotype was found (blue = United States, light red = Canada). The proportion of each color inside pie charts represents the relative frequency of the particular haplotype between the geographical regions. The ASVs were filtered, being considered real if present in at least four samples and with at least 30 reads in each sample. After filtering, the ASVs were plotted on a map, as pie charts with different colors representing the frequencies of the different ASVs as indicated in key using Leaflet (https://leafletjs.com) interactive maps to create geospatial visualization of anthelmintic resistance frequency patterns.

**Fig. 6:**
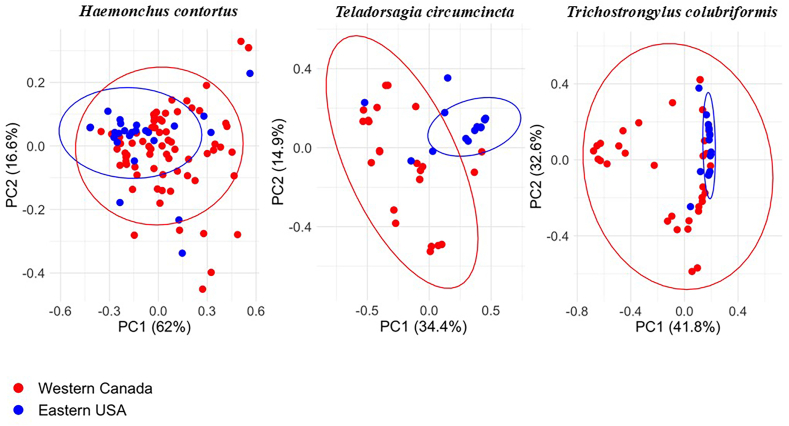
**Principal component analysis of the frequency of resistant ASVs between Eastern United States and Western Canadian samples.** The figure shows the Principal Component Analysis (PCA) plots generated for the *Haemonchus contortus, Teladorsagia circumcincta* and *Trichostrongylus colubriformis* populations from Eastern United States and Western Canadian samples. Samples are grouped based on the geographical location and the Bray-Curtis dissimilarity matrix was used to calculate the distance among the populations.

Isotype-1 β-tubulin ASVs were generated for each of the three nematode species from the amplicon sequence data from the 102 Western Canadian and 28 Eastern USA sheep flocks. The data was filtered to produce a final dataset that only included ASVs represented by at least 30 reads in at least four samples to minimize the risk of artifactual ASVs being considered and to focus on the major alleles (Supplementary Tables 1–3). This resulted in a final set of 13 (8 resistant, 5 susceptible), 23 (9 resistant and 14 susceptible), and 15 (8 resistant and 7 susceptible) ASVs for *H. contortus*, *T. circumcincta* and *T. colubriformis* respectively (Fig. 3). The ASV occurrence in individual flocks is shown in Fig. 4 and the geographical distribution of the ASVs carrying the F200Y (TTC > TAC) SNP for *H. contortus*, *T. circumcincta* and *T. colubriformis* in Fig. 5. Most ASVs carrying resistance SNPs are distributed across both Western Canada and Eastern USA with the two regions having a high degree of similarity overall, despite the discrepancy on sample numbers between the two regions. A Principal Component Analysis of individual flocks based on their resistance allele ASV composition also shows there is no clustering of flocks by country (Fig. 6).

## 4 Discussion

Benzimidazoles were the first truly broad-spectrum drug class to be used in GIN livestock parasite control (Brown et al., 1961; Gordon, 1961). They have been widely used in small ruminants for around 60 years, leading to widespread resistance in many ovine GIN species worldwide (Kaplan and Vidyashankar, 2012). They are also the drug class for which the molecular genetic basis of resistance is best understood providing an opportunity to investigate the molecular epidemiology of anthelmintic resistance in livestock parasites.

The extent to which anthelmintic resistance emerges independently in a region or depends on animal movement and the resultant translocation of resistant parasites is still poorly understood. In this study, we have investigated this question by undertaking a comparison of the benzimidazole resistance SNPs and alleles present in the major gastrointestinal nematode parasites in sheep flocks from two geographically distant regions of North America, Western Canada and Eastern USA. Benzimidazole resistance has been known to be widespread in small ruminant trichostrongylid nematodes in the USA for many years (Uhlinger et al., 1992; Howell et al., 2008; Grosz et al., 2013) and more recently in Canada (Barrère et al., 2013a; Falzon et al., 2013; Queiroz et al., 2020). There is significant small ruminant livestock movement across North America leading to the potential for the translocation of resistant parasites between the two countries. Overall, we found a high degree of similarity of the major benzimidazole resistance SNPs and alleles between the two regions for all three major parasite species: *H. contortus*, *T. circumcincta* and *T. colubriformis.* These results support a model of shared origins of resistance across the North American continent and the importance of animal movement in the emergence of resistance.

### 4.1 Similarity of canonical isotype-1 β-tubulin resistance SNPs in the major trichostrongylid nematode species in Western Canadian and USA sheep flocks

The overall frequencies of the canonical resistance SNP frequencies levels indicate the extent of benzimidazole resistance for the different major ovine parasite species in the two different regions of North America. For *H. contortus*, benzimidazole resistance SNPs are essentially at fixation in almost all flocks examined, with very few susceptible alleles present, revealing the advanced stage of benzimidazole resistance for this parasite species in both regions. This result is consistent with the propensity of this parasite species to develop benzimidazole resistance in many other parts of the world. In contrast, for *T. circumcincta* and *T. colubriformis* although the canonical resistance SNPs are at fixation at almost all flocks examined in Eastern USA, the prevalence and frequencies were much lower in the Western Canadian flocks. The less advanced stage of benzimidazole resistance for these two parasite species in Western Canada than in Eastern USA may reflect the lower level of benzimidazole anthelmintic usage in sheep parasite control in the former region.

There are a variety of canonical benzimidazole resistance SNPs that have been reported in different parts of the world for different ovine trichostrongylid nematode species. Namely, F200Y (TTC > TAC) (Kwa et al., 1994; Elard and Humbert, 1999), F167Y (TTC > TAC) (Silvestre and Cabaret, 2002), E198A (GAA > GCA) (Ghisi et al., 2007), E198L (GAA > TTA), E198V (GAA > GTA), E198G (GAA > GGA) (Francis et al., 2024; Francis and Šlapeta, 2023) E198K (GAA > AAA) (Mohammedsalih et al., 2020; Kalule et al., 2023), E198I (GAA > ATA) and E198∗(stop codon) (Mohammedsalih et al., 2020). Based on the available published global information to date, F200Y (TTC > TAC) is the most common overall, then F167Y (TTC > TAC) and then E198A (GAA > GCA) with the other 198 codon SNPs being less common. Importantly though, the prevalences of each of these SNPs can vary substantially between regions (Avramenko et al., 2019; Martínez-Valladares et al., 2020). In this study, it is notable that the canonical benzimidazole resistance SNPs present in each of the three major trichostrongylid nematode species were identical in the two geographically distant regions of North America. For *H. contortus*, F200Y (TTC > TAC) predominated and the F167Y (TTC > TAC) was also present at a moderate prevalence in both regions. No codon 198 SNPs were present in either region for this parasite species. In the case of *T. circumcincta*, F200Y (TTC > TAC) again predominated, but in this case F167Y (TTC > TAC) was absent and instead E198A (GAA > GCA) was present at low prevalence in both regions. For *T. colubriformis*, F200Y (TTC > TAC) was the only resistance SNP present with SNPs at codons 167 and 198 being absent in both regions. The identical pattern of the “benzimidazole resistance SNP profiles” of each parasite species is consistent with common origins of resistance for these two geographically distant regions. The higher frequency of resistance SNPs in the USA may suggest resistance has been established there for longer than in Canada. However, it is not possible to directly infer the direction of the spread of resistance from this dataset which is essentially a cross-sectional study.

### 4.2 Similarity of isotype-1 β-tubulin alleles in the major trichostrongylid species supports a hypothesis of shared origins of benzimidazole resistance in Western Canadian and USA sheep flocks

Haplotype network analysis has been previously applied to relatively small isotype-1 β-tubulin Sanger sequencing datasets from *H. contortus* and *T. circumcincta* populations from several other regions (Redman et al., 2015; Chaudhry et al., 2015, 2016). This has led to the hypothesis that benzimidazole resistance SNPs arise from a relatively small number of origins in a region/country and then spread by translocation of the resistant parasites through host movement (Gilleard and Redman, 2016; Chaudhry et al., 2015, 2020). The isotype-1 β-tubulin amplicon sequencing data produced in this study is of a much larger scale, both in terms of numbers of populations and sequencing depth, than those previous studies. Although the ASVs derived from amplicon sequencing are short (300–350 bp), the high genetic diversity of trichostrongylid nematodes allows some analysis of allelic diversity and phylogenetic relationships. Overall, the resistance SNPs were present on the same ASVs (resistance alleles) in the two geographically distant regions of North America for all three parasite species.

In the case of *H. contortus,* there were seven ASVs detected carrying the F200Y (TTC > TAC) SNP overall. Of these, three were present in both regions and at broadly similar prevalences and frequencies (Hc1, Hc2 and Hc3 (Fig. 4, Fig. 5). Hc9, Hc13 and Hc14, were present only in Western Canada but these were present at very low prevalence and frequencies. The F167Y (TTC > TAC) SNP was present on just a single ASV (Hc4) which was the same in both regions. In the case of *T. circumcincta* there were eight ASVs detected carrying the F200Y (TTC > TAC) SNP overall. Of these, five were present in both regions and at broadly similar prevalences and frequencies (Tc6, Tc7, Tc8, Tc15, Tc21) (Fig. 4, Fig. 5). Tc18, Tc20 and Tc42 were present only in Eastern United States at very low prevalence and frequencies. The E198A (GAA > GCA) SNP was present on just a single ASV (Tc14) which was the same in both regions. Finally, in the case of *T. colubriformis* the F200Y (TTC > TAC) SNP was present on a total of eight ASVs. Of these, including the most prevalent (Tco2 and Tco3), were present in both regions. Interestingly, one ASV (Tco7) was present in ten Canadian flocks and at a very high frequency on one but was absent from the Eastern USA flocks. This could represent a geographically independent resistance allele or may just reflect the more limited sample number for the Eastern USA.

### 4.3 Summary and conclusions

Deep amplicon sequencing of PCR-amplified resistance alleles provides a powerful approach to examine the distribution of anthelmintic resistance SNPs and alleles at a scale not previously possible. Analysis and geographical mapping of such data can potentially help us to understand the origins and spread of anthelmintic resistance. In this study, we have found that the major sheep gastrointestinal parasites in Western Canada and Eastern USA share the same benzimidazole resistance SNPs and share the same major resistance alleles at similar relative frequencies consistent with common origins. For two of the three parasite species examined, benzimidazole resistance is less advanced in Western Canada than Eastern USA which is consistent with the known patterns of anthelmintic use in the two countries. Consequently, we hypothesize that these shared resistance alleles may have originated in the USA under the influence of intense selection pressure due to frequent anthelmintic use and then subsequently spread to Canada by animal movement. This hypothesis has practical implications highlighting the dangers of animal importation from regions where anthelmintic resistance is already established, the relevance of biosecurity and the need for the adoption of appropriate quarantine procedures. Future population genomic studies are required to further test and refine this model of anthelmintic resistance emergence and help direct approaches to more sustainable parasite control in ruminant livestock.

## CRediT authorship contribution statement

**Camila Queiroz:** Writing – review & editing, Writing – original draft, Visualization, Validation, Project administration, Methodology, Investigation, Data curation, Conceptualization. **Michel Levy:** Writing – review & editing, Supervision, Funding acquisition, Conceptualization. **Russell Avramenko:** Writing – review & editing, Methodology, Investigation, Formal analysis. **Rebecca Chen:** Writing – review & editing, Methodology, Formal analysis. **Michaela Seal:** Formal analysis. **Elizabeth Redman:** Writing – review & editing, Supervision, Methodology, Formal analysis. **Anne Zajac:** Writing – review & editing, Methodology, Investigation. **John Stuart Gilleard:** Writing – review & editing, Supervision, Resources, Funding acquisition, Data curation, Conceptualization.

## Conflicts of interest

None.

## Data Availability

Isotype-1 β-tubulin sequences generated from Western Canadian and Eastern United States samples are publicly available on the Sequence Read Archive (SRA - PRJNA1178696).
