# Supplementary material for: Similar patterns of benzimidazole resistance alleles in ovine gastrointestinal nematodes from Western Canada and Eastern United States supports their shared origins and subsequent spread
Source: Int J Parasitol Drugs Drug Resist. 2025 Oct 7;29:100620. doi: 10.1016/j.ijpddr.2025.100620 (PMC12547256; doi:10.1016/j.ijpddr.2025.100620)
Supplement: Multimedia component 1 [file mmc1.docx]

| **Supplementary Table 1. Summary of amplicon sequence variants (ASVs) identified in *Haemonchus contortus* for the haplotype network analysis.** | | | | | | | | | | | | | | | |  |
| --- | --- | --- | --- | --- | --- | --- | --- | --- | --- | --- | --- | --- | --- | --- | --- | --- |
|  |  |  |  |  |  |  |  |  |  |  |  |  |  |  |  |  |
|  |  |  |  |  |  |  |  |  |  |  |  |  |  |  |  |  |
| **F200Y** | | | **Location** | | | | | **F167Y** | | | **Location** | | | | |  |
| **ASV ID** | **Total frequency** | **Total read number** | **AB** | **BC** | **MB** | **SK** | **USA** | **ASV ID** | **Total frequency** | **Total read number** | **AB** | **BC** | **MB** | **SK** | **USA** |  |
| **Hc1** | 49.76% | 229197 | 39 | 7 | 11 | 23 | 26 | **Hc4** | 4.90% | 22555 | 23 | 3 | 6 | 11 | 17 |  |
| **Hc2** | 32.19% | 148294 | 36 | 7 | 11 | 23 | 23 |  |  |  |  |  |  |  |  |  |
| **Hc3** | 8.42% | 38790 | 26 | 6 | 8 | 18 | 8 |  |  |  |  |  |  |  |  |  |
| **Hc9** | 0.29% | 1319 | 4 | 0 | 2 | 3 | 1 | **Susceptible** | | | **Location** | | | | |  |
| **Hc10** | 0.27% | 1229 | 7 | 1 | 1 | 8 | 9 | **ASV ID** | **Total frequency** | **Total read number** | **AB** | **BC** | **MB** | **SK** | **USA** |  |
| **Hc13** | 0.14% | 644 | 7 | 0 | 1 | 5 | 1 | **Hc5** | 1.69% | 7773 | 19 | 4 | 3 | 9 | 3 |  |
| **Hc14** | 0.13% | 590 | 5 | 0 | 1 | 1 | 0 | **Hc6** | 0.60% | 2758 | 5 | 1 | 1 | 0 | 2 |  |
| **E198A** | | | **Location** | | | | | **Hc7** | 0.31% | 1414 | 10 | 1 | 2 | 1 | 0 |  |
| **ASV ID** | **Total frequency** | **Total read number** | **AB** | **BC** | **MB** | **SK** | **USA** | **Hc12** | 0.19% | 896 | 7 | 0 | 3 | 1 | 1 |  |
| **Hc11** | 0.20% | 930 | 0 | 0 | 0 | 0 | 4 | **Hc15** | 0.11% | 508 | 3 | 0 | 1 | 1 | 2 |  |

| **Supplementary Table 2. Summary of amplicon sequence variants (ASVs) identified in *Teladorsagia circumcincta* for the haplotype network analysis*.*** | | | | | | | | | | | | | | | |  |
| --- | --- | --- | --- | --- | --- | --- | --- | --- | --- | --- | --- | --- | --- | --- | --- | --- |
|  |  |  |  |  |  |  |  |  |  |  |  |  |  |  |  |  |
| **F200Y** | | | **Location** | | | | | **Susceptible** | | | **Location** | | | | |  |
| **ASV ID** | **Total frequency** | **Total read number** | **AB** | **BC** | **MB** | **SK** | **USA** | **ASV ID** | **Total frequency** | **Total read number** | **AB** | **BC** | **MB** | **SK** | **USA** |  |
| **Tc6** | 6.65% | 5658 | 2 | 4 | 1 | 6 | 5 | **Tc1** | 10.81% | 9195 | 20 | 2 | 3 | 5 | 0 |  |
| **Tc7** | 6.10% | 5186 | 5 | 0 | 1 | 6 | 19 | **Tc2** | 9.45% | 8041 | 14 | 4 | 4 | 9 | 0 |  |
| **Tc8** | 2.80% | 2378 | 2 | 6 | 0 | 3 | 4 | **Tc3** | 8.66% | 7366 | 17 | 3 | 5 | 6 | 0 |  |
| **Tc15** | 1.72% | 1464 | 4 | 0 | 0 | 1 | 5 | **Tc4** | 6.94% | 5905 | 15 | 2 | 4 | 5 | 2 |  |
| **Tc18** | 1.49% | 1269 | 0 | 0 | 0 | 0 | 7 | **Tc5** | 6.92% | 5888 | 11 | 1 | 3 | 3 | 0 |  |
| **Tc20** | 0.87% | 744 | 0 | 0 | 0 | 0 | 8 | **Tc9** | 2.53% | 2150 | 13 | 3 | 4 | 6 | 0 |  |
| **Tc21** | 0.84% | 718 | 1 | 0 | 1 | 0 | 2 | **Tc10** | 2.43% | 2065 | 9 | 1 | 3 | 4 | 0 |  |
| **Tc42** | 0.31% | 260 | 0 | 0 | 0 | 0 | 5 | **Tc11** | 2.20% | 1874 | 6 | 0 | 1 | 1 | 0 |  |
| **E198L** | | | **Location** | | | | | **Tc13** | 1.95% | 1663 | 5 | 1 | 1 | 1 | 0 |  |
| **ASV ID** | **Total frequency** | **Total read number** | **AB** | **BC** | **MB** | **SK** | **USA** | **Tc16** | 1.64% | 1399 | 1 | 0 | 1 | 1 | 0 |  |
| **Tc14** | 1.83% | 1554 | 3 | 0 | 0 | 1 | 3 | **Tc17** | 1.54% | 1310 | 5 | 1 | 1 | 2 | 0 |  |
|  |  |  |  |  |  |  |  | **Tc22** | 0.81% | 693 | 1 | 1 | 0 | 2 | 0 |  |
|  |  |  |  |  |  |  |  | **Tc25** | 0.69% | 590 | 4 | 1 | 1 | 1 | 0 |  |
|  |  |  |  |  |  |  |  | **Tc34** | 0.41% | 346 | 4 | 0 | 1 | 3 | 0 |  |
|  |  |  |  |  |  |  |  | **Tc35** | 0.39% | 331 | 2 | 0 | 0 | 3 | 3 |  |

| **Supplementary Table 3. Summary of amplicon sequence variants (ASVs) identified in *Trichostrongylus colubriformis* for the haplotype network analysis*.*** | | | | | | | | | | | | | | | |  |
| --- | --- | --- | --- | --- | --- | --- | --- | --- | --- | --- | --- | --- | --- | --- | --- | --- |
|  |  |  |  |  |  |  |  |  |  |  |  |  |  |  |  |  |
| **F200Y** | | | **Location** | | | | | **Susceptible** | | | **Location** | | | | |  |
| **ASV ID** | **Total frequency** | **Total read number** | **AB** | **BC** | **MB** | **SK** | **USA** | **ASV ID** | **Total frequency** | **Total read number** | **AB** | **BC** | **MB** | **SK** | **USA** |  |
| **Tco2** | 26.18% | 41417 | 17 | 2 | 2 | 17 | 22 | **Tco1** | 27.59% | 43646 | 18 | 2 | 8 | 10 | 2 |  |
| **Tco3** | 17.69% | 27981 | 10 | 2 | 1 | 13 | 18 | **Tco4** | 7.54% | 11929 | 6 | 0 | 2 | 5 | 0 |  |
| **Tco7** | 1.97% | 3117 | 6 | 0 | 0 | 4 | 0 | **Tco5** | 4.35% | 6884 | 10 | 2 | 3 | 4 | 0 |  |
| **Tco9** | 1.49% | 2356 | 2 | 0 | 0 | 2 | 2 | **Tco6** | 3.71% | 5870 | 10 | 1 | 3 | 7 | 1 |  |
| **Tco13** | 0.55% | 866 | 5 | 0 | 1 | 5 | 11 | **Tco8** | 1.76% | 2780 | 6 | 1 | 1 | 3 | 0 |  |
| **Tco16** | 0.42% | 659 | 0 | 0 | 0 | 0 | 8 | **Tco10** | 1.23% | 1952 | 3 | 0 | 1 | 0 | 0 |  |
| **Tco17** | 0.39% | 613 | 5 | 0 | 0 | 6 | 2 | **Tco14** | 0.53% | 841 | 3 | 0 | 1 | 1 | 0 |  |
| **Tco27** | 0.13% | 212 | 2 | 0 | 0 | 2 | 0 |  |  |  |  |  |  |  |  |  |
